# Integrative analysis of immune molecular subtypes and microenvironment characteristics of bladder cancer

**DOI:** 10.1002/cam4.4071

**Published:** 2021-06-24

**Authors:** Jinlong Cao, Jianpeng Li, Xin Yang, Pan Li, Zhiqiang Yao, Dali Han, Lijun Ying, Lijie Wang, Junqiang Tian

**Affiliations:** ^1^ Department of Urology The Second Hospital of Lanzhou University Lanzhou People's Republic of China; ^2^ Key Laboratory of Urological Diseases of Gansu provincial Lanzhou People's Republic of China; ^3^ Reproductive Medicine Center The Second Hospital of Lanzhou University Lanzhou People's Republic of China; ^4^ Department of Gynecology The Second Hospital of Lanzhou University Lanzhou People's Republic of China

**Keywords:** decision tree model, immune molecular subtypes, immunotherapy, random forest, the cancer genome atlas

## Abstract

The emergence of immunotherapy has provided an option of treatment methods for bladder cancer (BC). However, the beneficiaries of immunotherapy are still limited to small‐scale patients, and immunotherapy‐related adverse events often occur. It is a major challenge for clinical work to study the immune subtypes of BC and the molecular mechanism of immune escape, and identify the immune responders accurately. Here, we explore the immune molecular subtypes of bladder cancer and potential escape mechanisms. First, we screened the expression profiles of 303 differentially expressed immune‐related genes in BC patients from the Cancer Genome Atlas (TCGA) database, and successfully identified 4 molecular subtypes of BC. By comparing the clinical characteristics, immune cells infiltration, the expression of checkpoint genes, human leukocyte antigen (HLA) genes, and gene mutation status of different subtypes, we identified different clinical and immunological characteristics of 4 subtypes. Among 4 subtypes, Cluster 2 met the general characteristics of immunotherapy responders and responded well to immunotherapy, while Cluster 4 had the highest expression of immune characteristics, and is similar to the immune environment of normal bladder tissue. Then, the weighted gene co‐expression network analysis (WGCNA) of immune‐related genes revealed that brown module was positively correlated with subtypes. Pathway enrichment analysis explored the major pathways associated with subtypes, which are also associated with immune escape mechanisms. Moreover, the decision tree model, which was constructed by the principle of random forest screening factors, was also validated in internal validation set and external validation set from the Gene Expression Omnibus (GEO) cohort (GSE133624), and could achieve accurate subtypes prediction for BC patients with high‐throughput sequencing. Taken together, we explored the immune molecular subtypes and their mechanisms of BC, and these results may provide guidance for the development of new BC immunotherapy strategies.

## Graphical abstract

We successfully identified 4 molecular subtypes of BC via TCGA database. Moreover, the establishment of decision tree model, by the principle of random forest screening factors, can achieve accurate subtype prediction for clinical BC patients with high‐throughput sequencing.

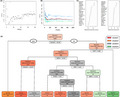

## 1 INTRODUCTION

Bladder cancer (BC) is ranked as the ninth most frequently diagnosed cancer worldwide. It is mainly represented by bladder urothelial carcinoma, which accounts for 90% of BC.^1^ According to the World Health Organization (WHO), there were 549,000 new cases and 200,000 deaths for BC worldwide in 2018.^2^ BC deaths will continue to increase in some low sociodemographic index countries in the next 10 years.^3^ The traditional treatments for BC mainly include surgical resection and chemotherapy, but there is a high distant metastasis and recurrence rate, the 5 years overall survival rate remains at 15%–20%.^4^ The emergence of immune checkpoint inhibitors has ended the deadlock of no significant progress in the treatment of BC after Bacillus Calmette–Guerin (BCG) for more than 30 years and increased treatment options for BC.^5^ But just like other solid tumors, the beneficiaries of immunotherapy for BC are still limited to small‐scale population, and tumor‐induced immune escape is a very common phenomenon.

The emergence of immunotherapy, especially immune checkpoint inhibitors, has revolutionized the treatment of many cancers. Currently, immune checkpoint inhibitors have been successfully used in the treatment of advanced bladder cancer and are increasingly being used in clinical work. Since April 2016, Food and Drug Administration (FDA) have approved atezolizumab, nivolumab, durvalumab, avelumab, and pembrolizumab, five PD‐1/PD‐L1 inhibitors for second‐line treatment options in patients with clinical advanced or chemotherapy resistant bladder cancer. Additionally, atezolizumab and pembrolizumab have replaced cisplatin as first‐line therapies for distant metastatic bladder cancer (stage Ⅳ). Many clinical studies have shown that bladder cancer immunotherapy is superior to conventional chemotherapy in terms of overall survival, progression‐free survival and objective remission rate.^6^ However, due to the high incidence of immune‐related adverse events (irAEs), the overall status of immunotherapy is not very ideal. Sharma et al.'s research indicated that objective response rate of advanced BC patients treated with nivolumab was 19.6%, and the effect of immunotherapy was not related to the expression of PD‐L1.^7^ Another study, which was published in the Lancet, found that objective response rate of BC patients via atezolizumab is different with the infiltration degree of immune cells, which is about 15%–27%.^8^ Moreover, the study indicated that the immune response of BC patients was associated with different immune subtypes, but specific subtypes analysis was not carried out in the study. If immunotherapy was used without selection, the overall response rate of treatment was lower, and the incidence of irAEs was 41.1%, far higher than the objective response rate of immunotherapy.^9^, ^10^, ^11^ Different from the side effects of traditional chemotherapy, irAEs are more serious in some cases, such as acute kidney injury, pancreatitis, and even death in some severe cases, with an incidence of about 3%.^12^, ^13^ The effect of immunotherapy is affected by the tumor immune microenvironment, and different patients show different therapeutic responses to immunotherapy, so there is marked individual variation in the clinical treatment outcome.^14^ In an era of personalized therapy, it is a major challenge for immunotherapy to identify the responders and nonresponders accurately. Moreover, the molecular mechanisms of immune subtypes and immune escape mechanisms in bladder tumor microenvironment are not entirely clear.

Most of the previous studies on immunotherapy about BC have focused on the identification of new immunotherapeutic targets and early clinical trials of immunotherapy efficacy for BC.^7^, ^8^, ^15^ The study of BC immune subtypes is at an early stage and no universally applicable model proposed. Currently, the common clinical markers for predicting immune response are mainly the expression of single immune checkpoint molecules, such as PD‐1, PD‐L1, and CTLA‐4. However, the expression of single immune checkpoint molecules has a limited predictive efficiency for immunotherapy responders, and the conclusions are inconsistent in several studies.^7^, ^16^ In recent years, many new predictive markers of immune response have been proposed, such as tumor mutation burden (TMB), somatic copy‐number alterations (SCNAs), microsatellite instability (MSI), T‐cell inflammatory microenvironment, and others.^17^ Despite the presence of multiple predictive markers, the complexity and heterogeneity of tumor immune microenvironment increase the difficulty of immunotherapy and affect the effectiveness of immunotherapy, and there are still no unified standard markers for clinical application.^18^ An accurate understanding of this heterogeneity contributes to the molecular subtypes of BC and the management of individualized therapy. Therefore, it is very necessary to thoroughly study the overall immune status, identify the immune molecular subtypes.

This study aims to comprehensively explore the heterogeneous immune molecular phenotypes of BC and its clinical significance. We screened the expression profiles of 303 differentially expressed immune‐related genes in BC patients from the TCGA database, and successfully identified 4 molecular subtypes of BC. By comparing the clinical characteristics, immune cells infiltration, the expression of checkpoint genes and HLA genes, and gene mutation status of different subtypes, we identified different clinical and immunological characteristics of 4 subtypes. Among 4 subtypes, Cluster 2 has a decreased immune profile, while Cluster 4 has the highest expression of immune characteristics, and similar to the immune environment of normal bladder tissue. Moreover, the establishment of decision tree model, by the principle of random forest screening factors, can achieve accurate subtype prediction for clinical BC patients with high‐throughput sequencing. These findings prove the feasibility of predicting immune responders via immune molecular subtypes, and provide guidance for the development of new BC immunotherapy strategies.

## 2 MATERIALS AND METHODS

### 2.1 Data acquisition and analysis

The mRNA‐seq data (counts format), simple nucleotide variation (SNV) data, clinical data of 409 BC patients were downloaded from the TCGA database (https://cancergenome.nih.gov/). The gene expression value of mRNA‐seq was log2‐transformed for further exploration. The external validation cohort GSE133624 included 36 BC samples and 29 para‐cancer samples with high‐throughput sequencing data were also downloaded from GEO database (www.ncbi.nlm.nih.gov/gds/). Then 1830 immune‐related genes were obtained from an immune gene set in the IMMport database (https://www.immport.org/resources). And 225 immune checkpoint gens and 19 human HLA genes were retrieved and obtained from National Center for Biotechnology Information website (www.ncbi.nlm.nih.gov/gene/). All statistical extraction and analyses were performed using R 3.4.0 (R Foundation for Statistical Computing) software.

### 2.2 Identification of BC subtypes based on the differentially expressed immune genes

The differentially expressed genes (DEGs) for 411 BC samples and 19 para‐cancer samples from TCGA were analyzed with the edgeR package, and |log_2_FC| >1.5 and *p* < 0.05 were set as the cutoff for DEGs. Veen algorithm was performed on the obtained DEGs and 1830 immune genes from the IMMport database, and obtained differentially expressed immune genes in BC. The ConsensusClusterPlus package^19^ was utilized to perform consistent clustering and screen of molecular subtypes based on the differentially expressed immune gene expression profiles. The optimal cluster number was determined by cumulative distribution function (CDF) curves of the consensus score. The immune genes from IMMport database with high expression in each subtype were identified using edgeR package and the cutoff was set |log2FC| >1.5 and *p* < 0.05. The top 100 upregulated genes in each subtype were selected and subjected to heatmap analysis and three‐dimensional principal component analysis (PCA) to distinguish different molecular subtypes. Moreover, Kaplan–Meier analysis and log‐rank test for overall survival were conducted for all upregulated immune genes in each subtype whose cutoff level was set at the median value of the expression value with the aid of survival package, and the most five prognosis‐related genes were displayed.

### 2.3 Clinical characteristics difference among four immune subtypes

The relationship between clinical characteristics and immune subtypes was analyzed and visualized. Kaplan–Meier curves and log‐rank tests were used to compare the overall survival and disease free survival of the 4 subtypes. Chi‐square test and one‐way ANOVA were used for the comparison of other clinical features, which include age, sex, TNM stage, high‐ and low‐grade, and clinic pathological stage.

### 2.4 Comparison of immune characteristics among four subtypes

Univariate Cox regression analysis was performed on 225 immune checkpoint genes in BC patients, and genes with *p* < 0.05 were selected. The differences of prognostic immune checkpoint genes among 4 subtypes were demonstrated in the form of heatmap via pheatmap package. Then, Kruskal–Wallis test was conducted to compare the differences of the 4 clinical common immune checkpoint genes (PDCD1, PDCD1LG2, CTLA4, and IDO1) and the 4 genes with the most significant differences among 4 subtypes (BACH2, LRRC32, SLFN11, and WWTR1), and ggplot2 package was used for the drawing of boxplot. Nineteen HLA genes were compared using heatmap and boxplot. The heatmap is drawn via pheatmap package, while the boxplot is drawn via ggpubr package.

Immune cells and immune‐related scores are important factors in the tumor immune microenvironment. We used MCPcounter^20^ R package and TIMER website (http://timer.cistrome.org/)^21^ to calculate and obtain scores of 10/8 types of immune‐related cells, respectively. The stromal score, immune score, and ESTIMATE score were achieved using estimate R package.^22^ The immune cells and immune‐related scores obtained were compared and demonstrated in the form of heatmap via pheatmap package.

### 2.5 Comparison of gene mutations among four subtypes

It is well known that the occurrence of cancer is the result of the accumulation of genetic mutations. TMB refers to a total number of coding errors, base substitutions, gene insertions, or deletions, etc. detected per million bases in somatic cells, and it is also considered to be a promising marker for predicting immune efficacy.^23^ First, we compared the overall mutation status of 4 subgroups of BC patients, including TMB of 4 subtypes, the number of mutated samples and the proportion of mutated genes. Then, the SNV data of each subtype patients were extracted. The Maftools package^24^ was used to analyze the overall states of gene mutations in each subtype, and the top 30 genes with the highest frequency of mutation in each subtype were shown in waterfall plots. The genes with mutation frequency greater than 8 in each subtype were shown in the form of word cloud plot via wordcloud2 package.

### 2.6 Co‐expression genes analysis and pathways analysis

Weighted gene co‐expression network analysis (WGCNA)^25^ is a comprehensive algorithm used to perform analysis of various aspects of weighted correlation networks. The immune genes with high expression in each subtype were selected for co‐expression analysis via WGCNA package. First, adjacency was calculated from the soft threshold power β, and the soft threshold power β is tested via function softConnectivity in WGCNA. Second, the expression matrix is converted to an adjacency matrix, and the frequency of different connectivity points in the adjacency matrix is analyzed. Third, the adjacency matrix is transformed into a topological matrix. Modules were detected through hierarchical clustering and dynamic tree cut function with the minimum number of genes was set at 30 per module, and then merged the modules with a height cut of 0.25. Fourth, we calculated and visualized the correlation between modules and clinical characteristics. Information on genes in each module was used for further analysis.

To explore the biological functions and pathways of gene modules, Kyoto Encyclopedia of Genes and Genomes (KEGG) enrichment analysis was performed using clusterProfiler package and the cutoff set as false discovery rate (FDR) <0.05 and count ≥4. The main associated pathways of each module were visualized by ggplot2 package. The Cytoscape 3.7.1^26^ was used to demonstrate the association of 6 modules with all enriched pathways.

### 2.7 Construction of decision tree model

WGCNA analysis suggested that the brown module was the most related module to BC subtype. We set 411 BC sequencing samples as training set, and selected the expression matrix (log2‐transformed) of 79 genes in the brown module as the characteristic variables of the model. Through the algorithm of random forest with setting mtrys as 5 and ntrees as 600, the random forest model was constructed via random Forest R package. Then, the rpart package was used to construct a decision tree model for the most important five genes, so as to predict the subtypes of clinical patients with sequencing.

### 2.8 Internal validation and external validation of the four subtypes

We randomly selected 30% (123) samples from 411 BC mRNA expression profiles as an internal validation set. The confusion matrix of the predicted results is displayed and evaluated the internal stability of the decision tree model. There were 36 BC samples and 29 para‐cancer samples with high‐throughput sequencing data in GSE133624, and highly consistent with the training set for its high‐throughput sequencing. The decision tree model was used to predict subtypes of 65 samples. TIMER website and estimate package were used to calculate the contents of 8 immune cells and immune‐related scores of the 36 tumor samples in GSE133624, and HLA genes expression profiles were also extracted. All of these features were shown in the form of heatmap via pheatmap R package, and compared with the immune characteristics of the original training set subtypes to test its predictive efficacy.

## 3 RESULTS

### 3.1 Identification of BC subtypes based on the differentially expressed immune genes

**FIGURE 1:**
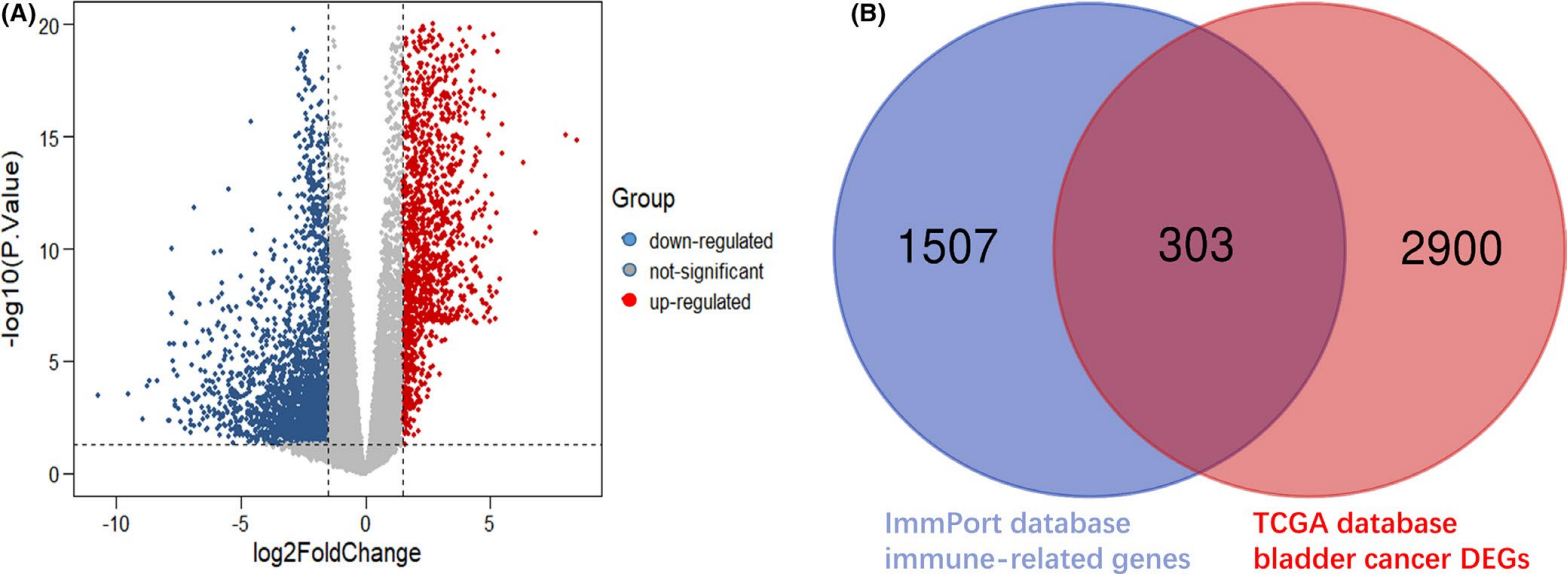
Screening the differentially expressed immune genes of bladder cancer. (A) Volcano plot of the distribution of differentially expressed genes in bladder cancer. Red/blue symbols classify the upregulated/downregulated genes according to the criteria: |log2FC| > 1.5 and *p*‐value < 0.05. (B) Veen calculation was performed for the differentially expressed genes in bladder cancer and the 1830 immune‐related genes obtained from the IMMport database, and to obtain 303 differentially expressed immune genes in bladder cancer

**FIGURE 2:**
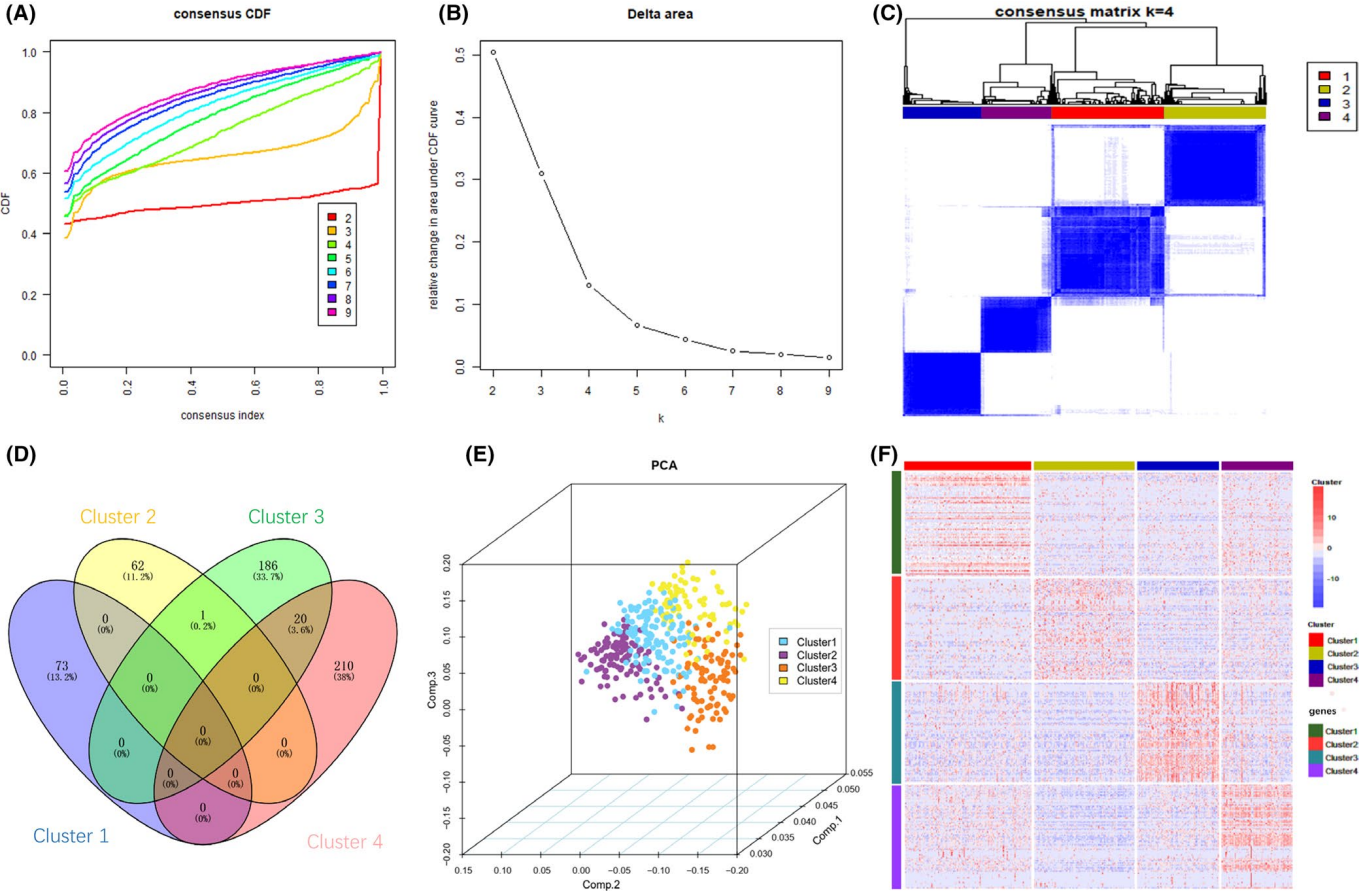
Identification of BC immune subtypes based on 303 differently expressed immune genes. (A) Consensus clustering cumulative distribution function (CDF) for k = 2–9. Different colors reflect different cluster numbers, the horizontal axis represents the consensus index, the vertical axis stands for CDF. (B) Relative change in area under CDF curve for k = 2–9. (C) Heatmap of sample clustering at consensus k = 4. (D) Intersection Venn diagram of significant high expression immune genes of 4 subtypes. (E) Three‐dimensional principal component analysis (PCA) according to the expression profiles of the top 100 significant high expression genes. (F) Heatmap of the top 100 significant high expression genes in 4 subtypes. Red represents high expression, and blue represents low expression

**FIGURE 3:**
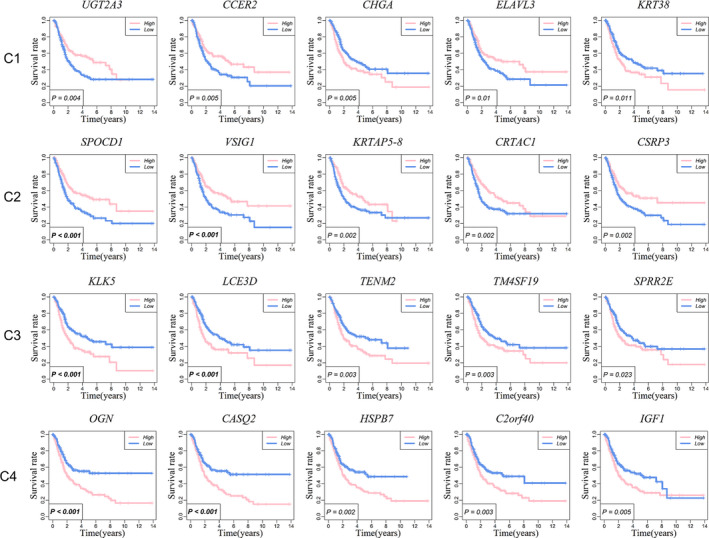
Kaplan–Meier analysis of overall survival for main DEGs of each subtype. Kaplan–Meier survival analysis and log‐rank test for the upregulated DEGs of each subtype were conducted, and the most five prognosis‐related genes were displayed. The 4 rows are Cluster 1, Cluster 2, Cluster 3, and Cluster4, respectively

Analysis of DEGs (Figure 1A) indicated that there were 3203 DEGs in BC and para‐cancer. Veen calculation (Figure 1B) result showed a total of 303 differentially expressed immune genes (Table S1). The 303 genes expression profiles were used to explore the immune subtypes of BC via ConsensusClusterPlus package. The optimal division was reached when k = 4 based on the CDF curves of the consensus score (Figure 2A,B). The 411 tumor samples were classified into 4 molecular subtypes underlying the 303 immune gene expression profile (Figure 2C). Among the 1830 immune‐related genes, 73 genes in Cluster 1, 63 genes in subtype Cluster 2, 207 genes in Cluster 3, and 230 genes in Cluster 4 were significantly upregulated (Figure 2D). More importantly, there are 552 genes expressed upregulated in each subtype, and only 1 gene overlapped in cluster 2 and cluster 3. Therefore, each subtype has relatively independent immune genes, and with significant differences among subtypes. Then, the top 100 upregulated genes in each subtype were extracted to construct PCA (Figure 2E) and heatmap (Figure 2F), which also showed a distinct expression pattern in the immune upregulated gene profiles of each subtype. Furthermore, we made Kaplan–Meier survival analysis and log‐rank test for the DEGs of 4 subtypes, the most significant results are shown in Figure 3. These genes can be seen as prognostic‐related gene markers of each subtype.

### 3.2 Clinical characteristics of the four subtypes

**FIGURE 4:**
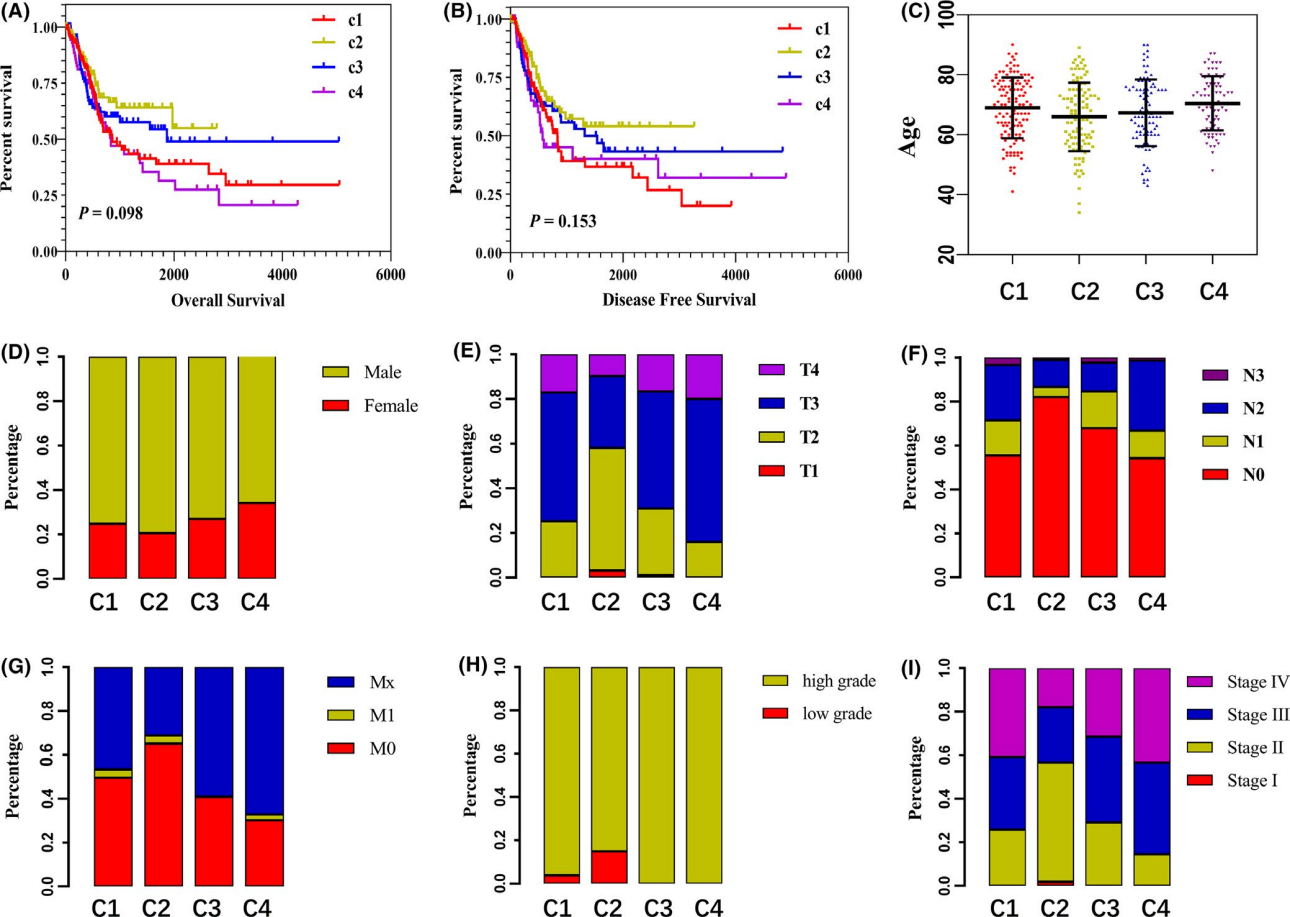
Relationship between 4 immune subtypes and clinical characteristics. (A, B) K‐M curves showing overall survival and disease‐free survival in patients with 4 subtypes, respectively. Different colors represent different subtypes. The *p*‐value was calculated using the log‐rank test by comparing 4 subtypes. (C) The age distribution of each subtype of patients. (D–I) Distribution ratio of gender, TNM stage, high‐ and low‐grade, and clinic pathological stage of each subtype, respectively

To explore the relationship between the BC clinical features and different subtypes, some clinical characteristics, including age, sex, TNM stage, high‐ and low‐grade, and clinic pathological stage, were analyzed and shown in Table S2. Figure 4A,B shows significant differences in overall and disease‐free survival among the 4 subtypes, among which Cluster 2 patients have a good survival prognosis, while Cluster 4 patients have the poorest prognosis. Figure 4C–I indicates that among the 4 subtypes, the cluster 2 patients' age was lower, and the TNM stage and clinicopathological stage were earlier, and most of low‐grade BC patients are also in Cluster 2. The clinical characteristics of Cluster 4 are the opposite of Cluster 2.

### 3.3 The difference of immune characteristics among four subtypes

**FIGURE 5:**
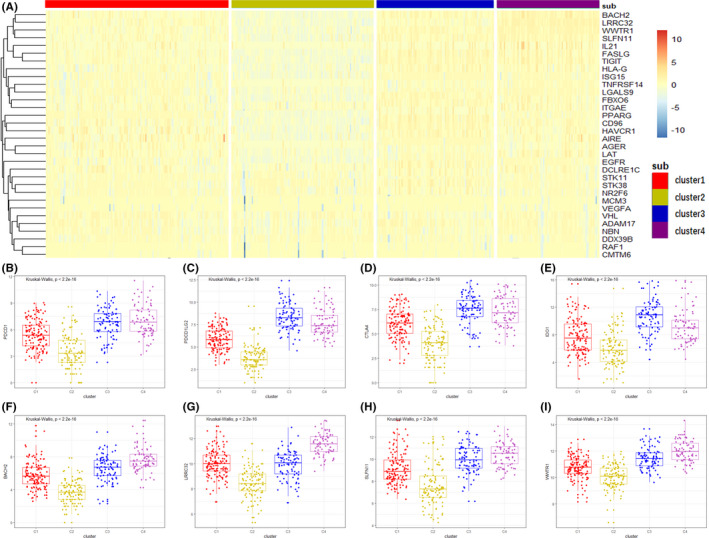
The expression difference of immune checkpoint genes in 4 subtypes. (A) Heatmap shows the expression levels of 32 prognostic immune checkpoint genes among 4 subtypes. (B–E) Comparison of 4 common immune checkpoint genes among 4 subtypes, namely PDCD1, PDCD1LG2, CTLA4, IDO1. Gene expression were log2‐transformed and P value is the result of Kruskal–Wallis test. (F–I) Comparison of 4 checkpoint genes with the most significant differences among 4 subtypes, namely BACH2, LRRC32, SLFN11, and WWTR1

Immune checkpoint genes are currently the target genes in clinical immunotherapy. First, univariate Cox regression analysis was performed on the relationship between 225 immune checkpoint genes and prognosis of BC patients. The results were shown in Table S3, and 32 immune checkpoint genes with *p* < 0.05 were selected for further study. Figure 5A shows that these genes are low expressed in Cluster 2 and high expressed in Cluster 3 and Cluster 4. Figure 5B–E shows the expression difference of 4 clinical common immune checkpoint genes, namely PDCD1, PDCD1LG2, CTLA4, and IDO1. Figure 5F–I shows the 4 genes (BACH2, LRRC32, SLFN11, and WWTR1) with the most significant differences among the 4 subtypes. These immune checkpoint genes were all low expressed in Cluster 2 and relatively high expressed in Cluster 3 and Cluster 4.

**FIGURE 6:**
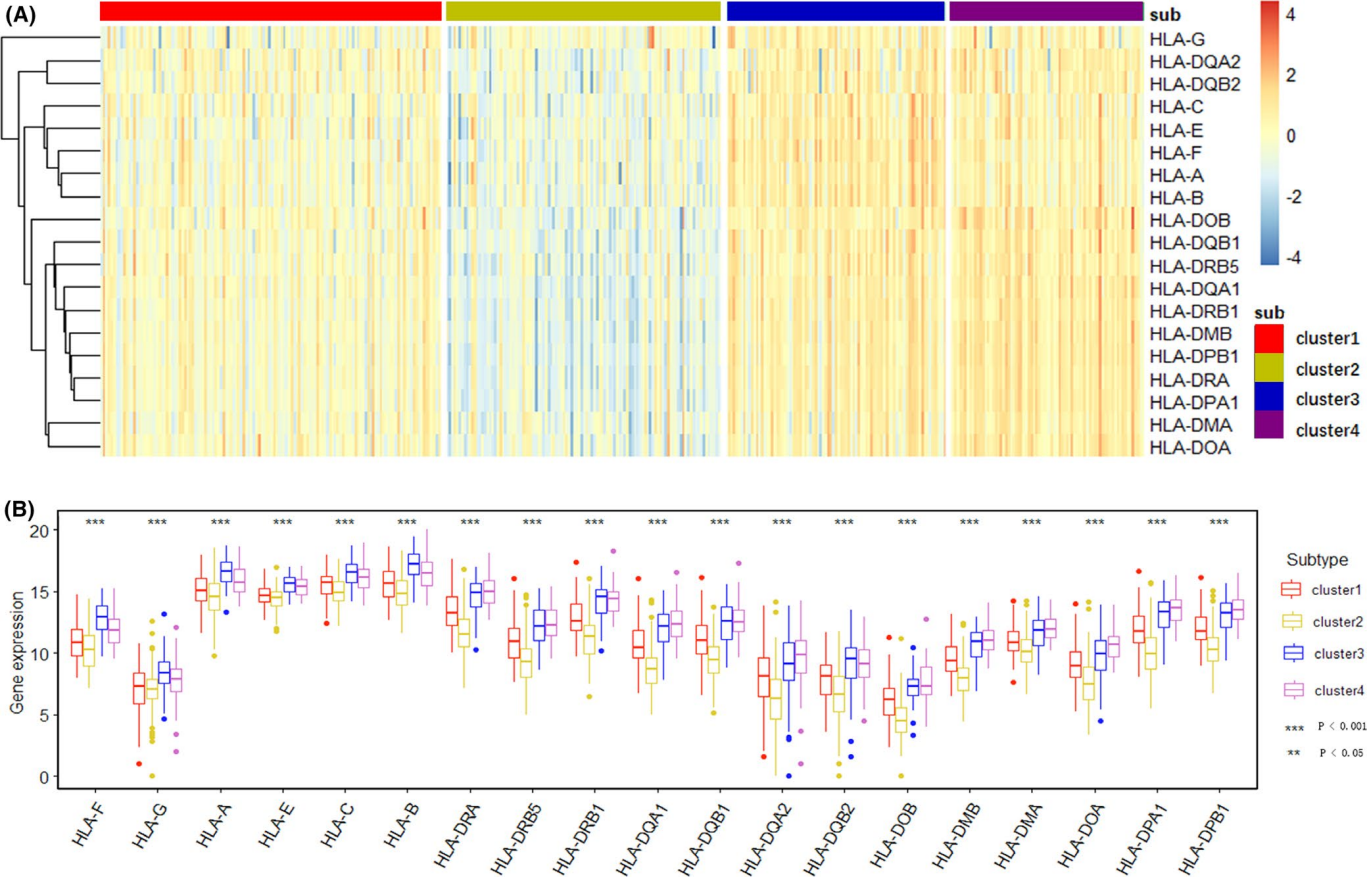
The expression difference of HLA genes in 4 subtypes (A) Heatmap shows the expression levels of 19 HLA genes among 4 subtypes. Red indicates high expression and blue indicates low expression. From left to right are Cluster 1, Cluster 2, Cluster 3, and Cluster 4. (B) Comparison of 19 HLA genes among 4 subtypes in the form of boxplot. The horizontal axis represents different genes and subtypes, and the vertical axis represents the amount of gene expression (log2‐transformed)

HLA genes are important immune genes in human body, and mainly involved in the immune response as the presenting molecules of endogenous and exogenous antigens. Tumor‐induced immune escape could alter the expression of HLA genes, allowing the tumor to evade the immune system without being killed.^27^ Figure 6A,B shows the difference of 19 HLA genes among the 4 subtypes in the form of heatmap and boxplot, respectively. Same as immune checkpoint genes, the expression of HLA genes in Cluster 2 is low, in line with the immunosuppressive subtype, and the immune escape occurs in tumor tissue, while the expression was high in Cluster 4.

**FIGURE 7:**
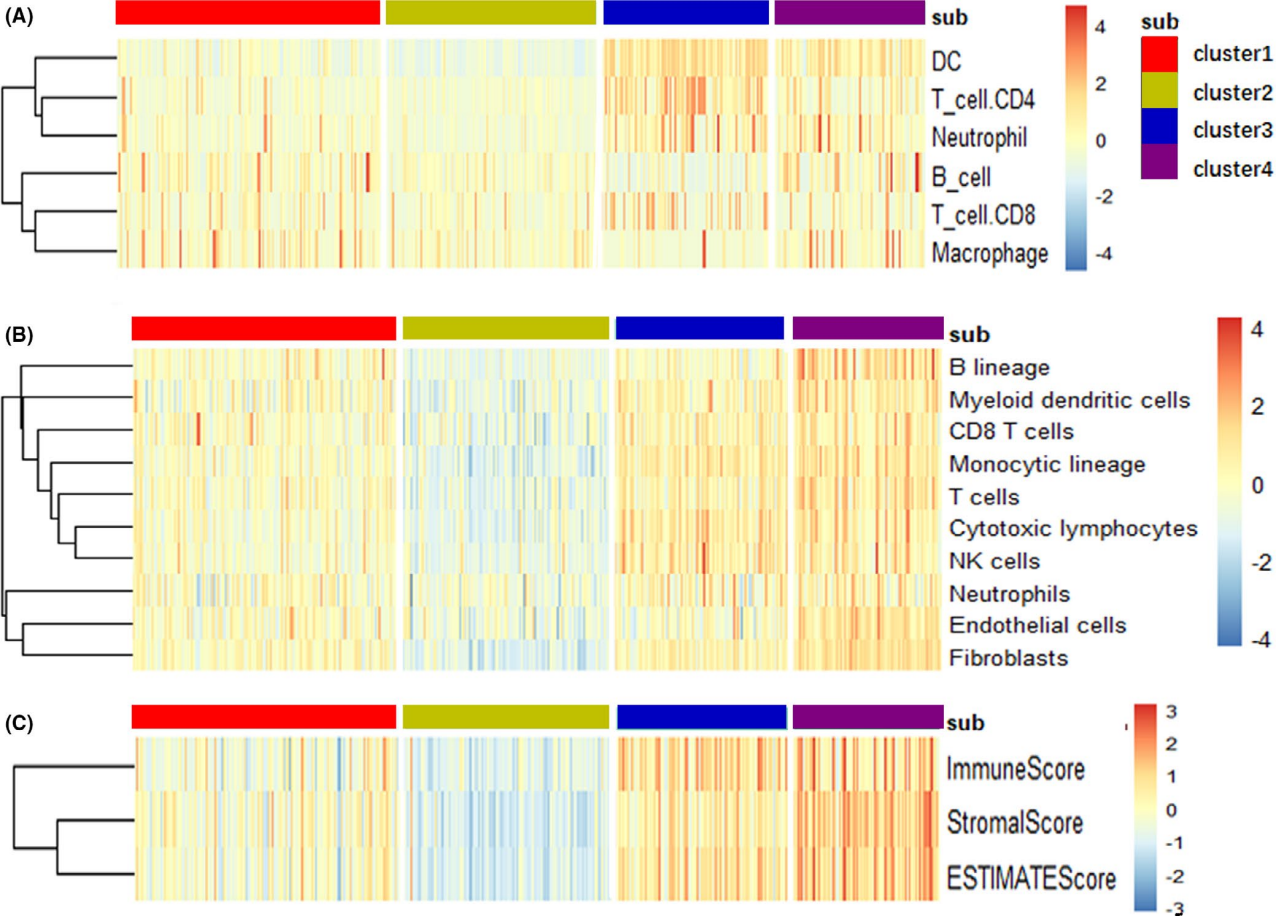
Different infiltration of immune cells in the 4 molecular subtypes of bladder cancer. (A) Expression scores of 6 immune cells obtained via TIMER website in the 4 subtypes of bladder cancer. Heatmap shows the associated gene expression value, with red indicating high expression and blue indicating low expression. (B) Gene expression scores of 10 immune cells obtained via MCPcounter calculation in 4 subtypes of bladder cancer. (C) The tumor stromal scores, the immune scores and the ESTIMATE scores in 4 molecular subtypes of bladder cancer

As for immune cells and immune scores, MCPcounter, TIMER and estimate three algorithms were used to calculate and obtain immune cells and immune scores in BC samples from TCGA cohort. These features were shown as heat maps in Figure 7A–C. Among them, the contents of various immune cells, stromal score, immune score, and ESTIMATE scores in Cluster 2 subtype are low expression, and in Cluster 4 are high expression.

In summary, most of the immune signatures are downregulated in subtype Cluster 2 and upregulated in subtype Cluster 4 comparison with the other subtypes, which suggests that Cluster 2 had a decreased immune profile and Cluster 4 had an enhanced immune profile. It also suggests that the immune characteristics in bladder cancer patients are increased or decreased synchronously.

### 3.4 The difference of gene mutations among four subtypes

**FIGURE 8:**
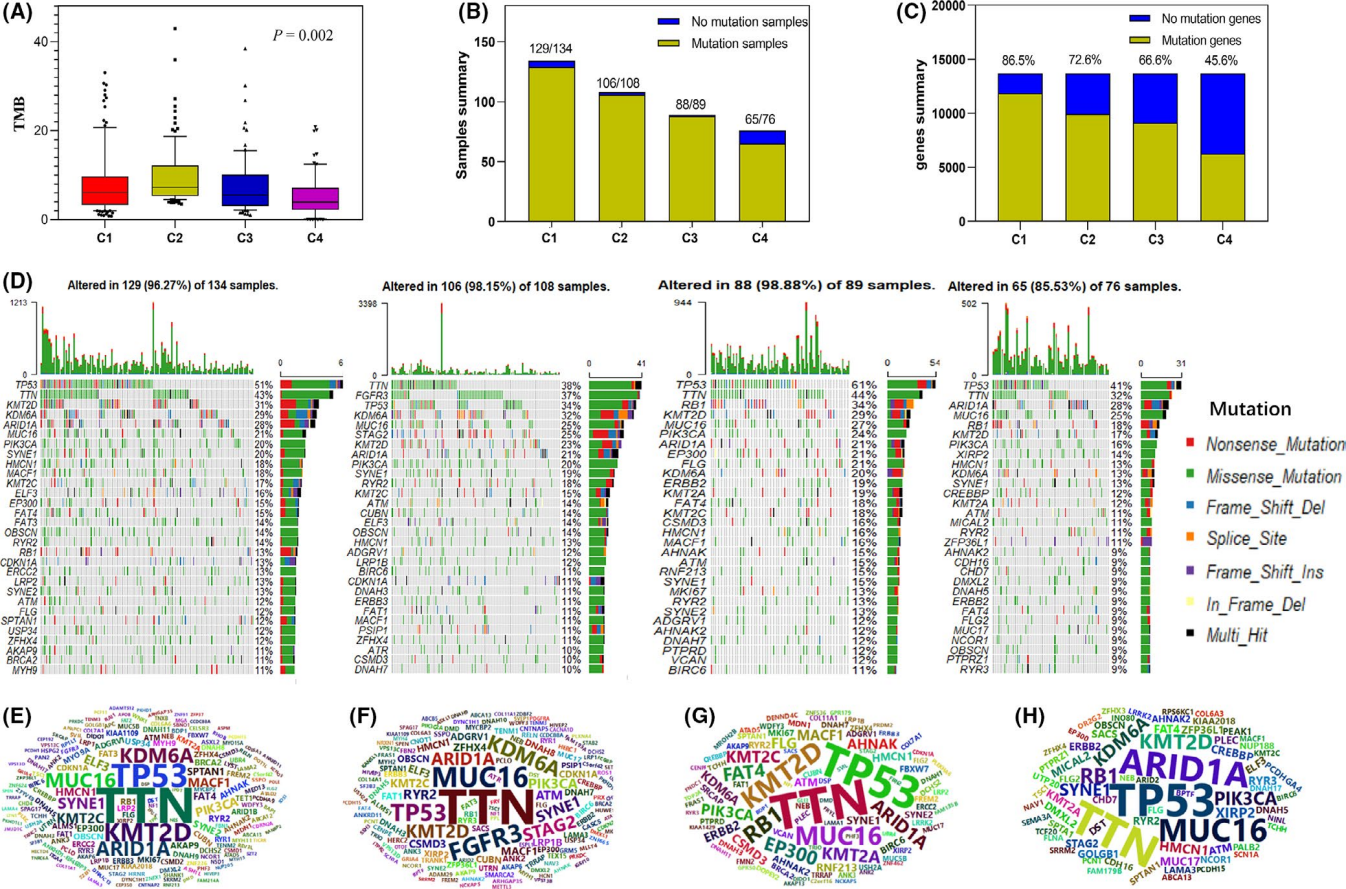
Comparison of genes mutations among 4 subtypes. (A) Comparison of tumor mutation burden (TMB) among the 4 subtypes, and *p*‐value is the result of one‐way ANOVA. (B) The number of mutated samples in 4 subtypes of bladder cancer. (C) The proportion of the total number of mutated genes in all transcriptome genes in 4 subtypes of bladder cancer. (D) The waterfall map shows the mutation distribution of the top 30 genes with mutation frequency in the 4 subtypes. (E–H) The word cloud plot represents the genes with a frequency greater than 8 in each subtype. The size of the gene names indicates the frequency of the gene mutations. From left to right are Cluster 1, Cluster 2, Cluster 3, and Cluster 4

The higher the TMB, the higher the gene mutation frequency of tumor cells, and the more tumor antigens carried on the cell surface and vulnerable to attack by the body's immune system, and the better the efficacy of immunotherapy could be achieved.^23^ As shown in Figure 8A, Cluster 2 has the highest TMB and may have a better immunotherapy effect. Figure 8B,C shows the total number of samples and proportions of mutation genes among the 4 subtypes, respectively. Cluster 4 has the fewest total number of mutated samples and the proportion of mutated genes. In addition, Figure 8D shows the top 30 genes of mutation frequency in the 4 subtypes in the form of waterfall map. Figure 8E–H shows the main mutation genes with a mutation frequency greater than 8. From these figures, it can be found that the mutation patterns of the 4 subtypes are different. TNN and FGFR3 mutation frequency are the highest in Cluster 2, while P53 mutation is dominant in other subtypes.

### 3.5 Co‐expression analysis and KEGG pathways analysis

**FIGURE 9:**
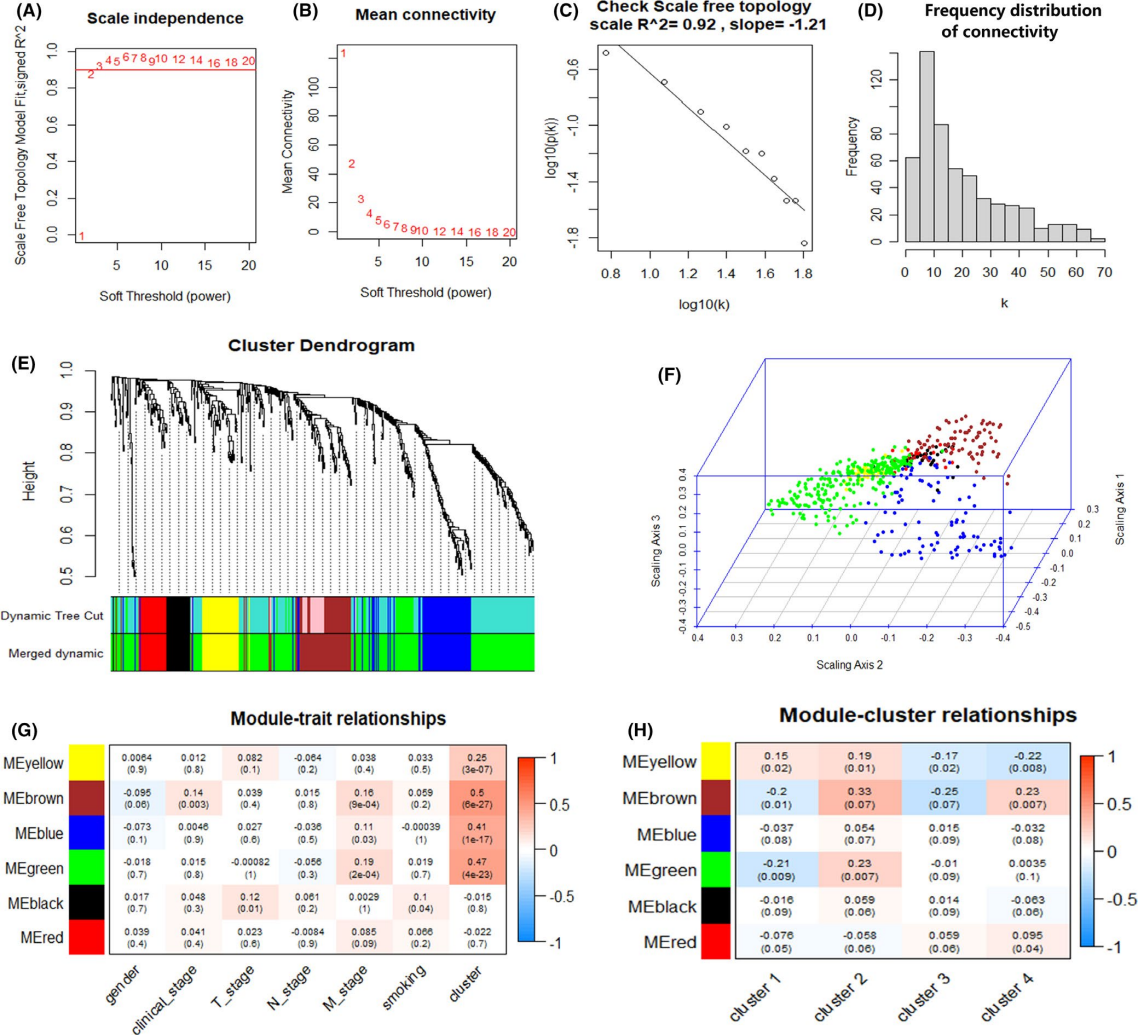
Weighted gene co‐expression network analysis (WGCNA) of high expression immune genes of 4 subtypes in TCGA cohort. (A) Analysis of the scale‐free fit index for various soft threshold powers β. (B) Analysis of the mean connectivity for various soft threshold powers. (C) Checking the scale free topology when β = 2. (D) Histogram of connectivity distribution when β = 2. (E) Hierarchical cluster analysis was conducted to detect co‐expression gene modules with corresponding color assignments. Different colors represent different modules. (F) The three‐dimensional map shows the distribution of genes in the six modules in cubical space. (G) Heatmap showing the correlation between feature vectors of six modules and clinical characteristics. (H) Heatmap showing the correlation between feature vectors of six modules and 4 BC subtypes

To explore the potential biological pathways associated with BC immune subtypes and immune escape, WGCNA, and KEGG enrichment analysis were conducted. To construct a scale‐free network, the soft threshold power β was set as 2 (Figure 9A,B). Figure 9C shows that logarithm log(k) of the node with the connection degree k is negatively correlated with the logarithm log(P[k]) of the probability of the node, and the correlation coefficient is 0.92. Figure 9D shows the frequency of different connectivity points in the adjacency matrix. Finally, a total of 6 modules with all immune‐related differentially expressed genes were identified and each module was assigned with a unique color as showed in Figure 9E. Each module and its corresponding number of genes was displayed in Table 1, and the specific genes within six modules obtained were displayed in Table S4. With the value of dissTOM as the coordinate, the positions of genes of each module in the three‐dimensional space are displayed as Figure 9F. The 552 immune‐related genes were clearly divided into 6 modules, and the genes within each module were consistent. Then, the correlation between each module and clinical characteristics (Figure 9G), and subtype (Figure 9H), was analyzed and visualized. According to the results, the brown module is the most correlated module with immune subtypes.

**TABLE 1:** The number of co‐expression genes of 6 modules

Modules	Genes
Black	32
Blue	94
Brown	79
Green	263
Red	35
Yellow	49

**FIGURE 10:**
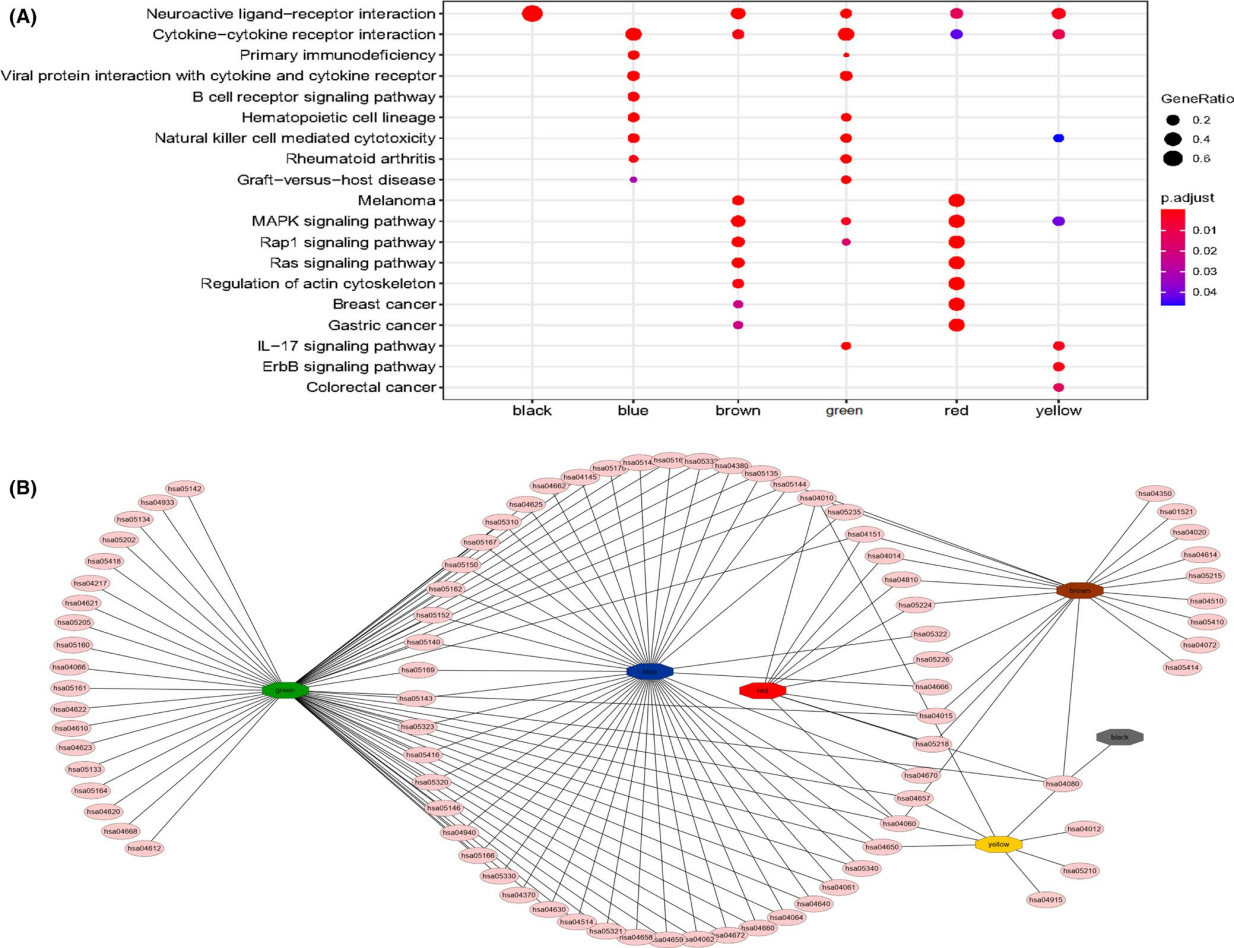
KEGG pathways analysis with co‐expressed genes in the 6 modules. (A) Main interaction pathways of the 6 modules genes. The size of the point represents the proportion of genes enriched in the specific pathway in each module genes, and the color represents the *p*‐value of enrichment analysis. (B) Cytoscape software presents the all enriched pathways associated with co‐expressed genes in the 6 modules. The pale red ellipsoids represent different enrichment pathways and the hexagons with different colors represent different co‐expressed gene modules

Subsequently, KEGG enrichment analysis of each module genes showed that the interaction pathways in each module (Figure 10A) were mainly enriched in neuroactive ligand‐receptor interaction, cytokine‐cytokine receptor interaction, primary immunodeficiency, viral protein interaction with cytokine and cytokine receptor, natural killer cell‐mediated cytotoxicity, graft‐versus‐host disease, regulation of actin cytoskeleton, and MAPK, Rap1, Ras, IL‐17, ErbB, B‐cell receptor, and other signaling pathways. These pathways, which are associated with the immune escape of BC, play an important role in the transformation of different subtypes of BC. The relationship network of enriched pathways in these modules was visualized as Figure 10B, and showed that blue module genes and green, red, brown, and yellow module genes have many common pathways. In addition, the red module and brown module also have more common pathways. These modules may share similar regulatory processes in the 4 subtypes.

### 3.6 Construction of decision tree model

**FIGURE 11:**
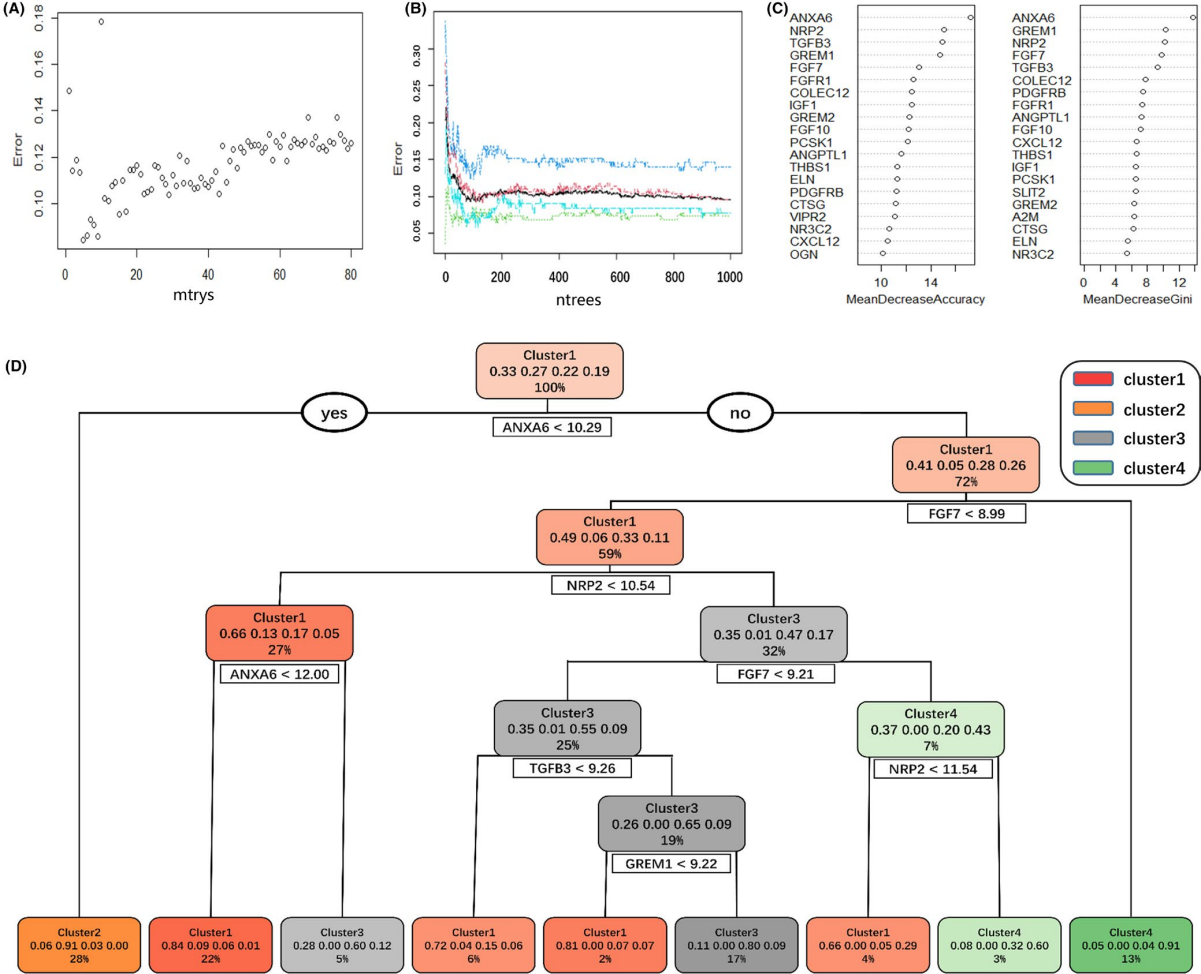
The construction of random forests and decision tree model. (A, B) The selection of two major parameters (mtry, ntrees) of the random forest model construction. When mtry set 5, the error of the model is the smallest, so the best decision tree model should include 5 variables. And ntrees >600, the model has high stability. (C) The top 20 most important genes ranked by mean decrease accuracy and mean decrease gini in the random forest model. (D) Optimal decision tree model in random forest. Here, each step is classified according to different gene expressions; In each rectangle, the first column is the subtype with the largest proportion in this part; The second column shows the proportion of the 4 subtypes in this part; The third column is the proportion of this part in the total samples

In order to make the subtypes available for clinical work, we constructed a decision tree model using 79 genes in the brown module. According to Figure 11A, when mtry set as 5, the model has a low error. Figure 11B shows that when ntrees set as 600, the model tends to stabilize. Therefore, the random forest model is constructed by using the above set values. Figure 11C shows the top 20 most important genes ranked by mean decrease accuracy and mean decrease gini in the random forest model. Figure 11D is the most accurate decision tree model in the random forest, which can be used to predict the immune subtypes of BC patients with sequencing.

### 3.7 Internal validation and external validation of the four subtypes

**FIGURE 12:**
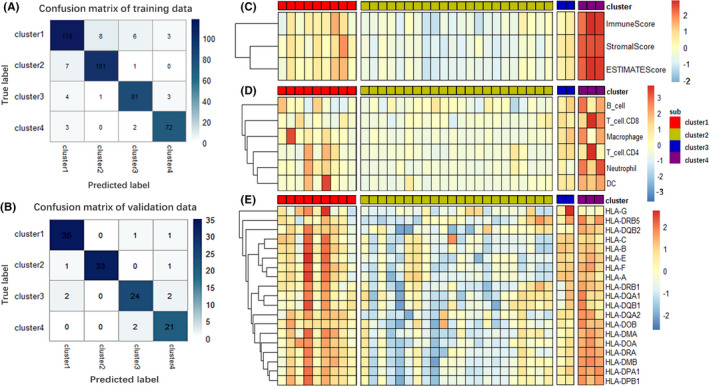
Internal validation and external validation. (A) The confusion matrix of the training set. The abscissa represents the correct subtype samples number, and the ordinate represents the subtype samples number predicted by the decision tree model. (B) The confusion matrix of the internal validation set. (C) Expression score of the tumor stromal scores, the immune scores and ESTIMATE score were calculated via estimate R package in 4 predicted molecular subtypes of GSE133624. Heatmap shows the associated expression value, with red indicating high expression and blue indicating low expression. (D) Expression scores of 6 immune cells obtained via TIMER website. (E) HLA genes express differently in the 4 predicted subtypes

Figure 12A presents the confusion matrix in the training sets of 411 BC samples, with an accuracy of 90.5%. And Figure 12B is the confusion matrix of the internal validation set, with an accuracy of 91.8%, which indicates that the model has high fitting ability in the internal samples. External validation was performed on the 36 BC samples from GSE133624, of which Cluster 1/2/3/4 has 9/22/2/3 samples, respectively (Table S5). By comparing various immune features in the external validation set (Figure 12C–E), it could be found that the differences of immune features between the external validation set and the training set were highly consistent. Therefore, the model has good stability and applicability. Notably, both the training set and the external validation set are high‐throughput sequencing samples. Additionally, we made subtype predictions for 29 para‐cancer samples in GSE133624 via the decision tree model, and 24 of them belonged to Cluster 4. For Cluster 4 has the highest expression of immune characteristics among all subtypes, it can be generally considered that normal bladder tissues have higher expression of immune characteristics. By comparing the immune characteristics of 4 subtypes, we believe that the immune subtype of normal bladder tissue is mostly Cluster 4, and different levels of immune escape and immune attenuation occur in the carcinogenesis of BC.

## 4 DISCUSSION

BC is the most common tumor of the urinary system, and its treatment progress has been slow. In recent years, the introduction of immune checkpoint inhibitors has provided a treatment option for BC. The identification of BC immune subtypes can classify the heterogeneous tumor microenvironment of BC, which is helpful for the biological research of BC and individualized immunotherapy. The study analyzed the heterogeneous BC microenvironment subtypes and related clinical significance systematically using public data extracted from the TCGA cohort. Four immune subtypes were found to exhibit significantly different clinical characteristics, immune escape mechanisms, genomic alterations, and clinical outcomes. Finally, these subtypes can be well identified by the decision tree model and external validation in the GEO database. For clinical patients with bladder cancer, high‐throughput sequencing, and immune molecular subtypes prediction can be performed, and Cluster 2 patients can be treated with immunotherapy for a better immune response. This study provides new ideas and strategies for bladder cancer immunotherapy, and to some extent, proves the feasibility of predicting immune responders via immune molecular subtypes.

Previous studies have analyzed BC subtypes based on immune cell expression in the immune environment.^28^ However, its clinical practice value is limited because of the difficulty in determining cells content. Moreover, the BC subtypes based on its pathological characteristics are related to immunotherapy checkpoint genes expression, but it could not accurately distinguish the complex microenvironment subtypes of BC.^29^ In this study, 4 immune molecular subtypes of BC were identified. Moreover, the decision tree model, which constructed by the principle of random forest screening factors, can achieve accurate prediction for subtypes of BC patients with high‐throughput sequencing. Through external validation of samples from GSE133624, we found that 24 of 29 para‐cancer samples belonged to Cluster 4 in the decision tree model prediction results. Although Cluster 4 had higher immune characteristics expression compared with other subtypes, it may be called the immune normal subtype, and Cluster 1, Cluster 2, and Cluster 3 occur varying degrees of immune attenuation. This indicates that there are multiple mechanisms of immune escape and transformation of multiple immune subtypes during the development of BC. Additionally, it also indicates that it may be unreasonable to call the subtype with high immune characteristics as the immune‐enhanced subtype in some subtypes‐related studies due to the lack of comparison with normal adjacent tissues.^30^, ^31^

The clinical characteristics of 4 immune subtypes were significantly different. For both overall survival and disease‐free survival, Cluster 2 patients have the best prognosis, while Cluster 4 subtypes had a poor prognosis. Compared with other subtypes, Cluster 2 patients tend to have lower age, early TNM stage, and clinicopathological stage. And most of the low‐grade BC patients are Cluster 2. The clinical characteristics of Cluster 4 are the opposite of Cluster 2. Cluster 2 patients have low‐expression immune characteristics and a better prognosis than other subtypes with higher immune characteristics. This is contrary to the prognosis of the immunodeficiency subtypes of ovarian cancer and hepatocellular carcinoma,^30^, ^31^ suggesting that the attenuation of immune characteristics of BC may be a potential mechanism of self‐protection.

To explore the immune‐related characteristics difference among 4 subtypes, we analyzed and compared differences in immune checkpoints, HLA genes, immune cells, and immune‐related scores among the 4 subtypes. These immune checkpoint genes and HLA genes are all low expressed in Cluster 2 and high expressed in Cluster 3 and Cluster 4. The algorithms MCPcounter, TIMER and estimate were used to estimate common immune cells and immune‐related scores. Heatmap comparison results show that Cluster 2 is an immune reduced subtype, while Cluster 4 was immune normal subtype. The occurrence of immune escape in BC is not only the change of immune checkpoint genes expression, but also the synchronous change of many immune characteristics. This characteristic is consistent with other solid tumors.^28^, ^30^, ^31^ There may exist a possible immune escape‐driven mechanism, similar to the tumor‐driven mechanism, that causes different patients to convert to different immune subtypes.

BC is a tumor with a large number of gene mutations, particularly in chromatin regulatory genes, which occur more frequently than other common cancer.^32^ TMB is an important indicator of gene mutation, and some studies have shown the TMB as a predictive biomarker for immunotherapy due to its refection of the overall neo‐antigens load.^33^, ^34^ Among the 4 subtypes, Cluster 2 had the highest TMB and may have a good immunotherapeutic effect. Meanwhile, Cluster 4 had a low TMB and the least total number of mutated genes and the proportion of mutant samples. The waterfall map and word cloud map were used to analyze the main gene mutation landscapes in 4 subtypes, and it was found that the gene mutation patterns of 4 subtypes were significantly different. TNN and FGFR3 mutation frequency are the highest in Cluster 2, while P53 is dominant in other subtypes. TNN, FGFR3, and P53 are common mutated genes in BC.^35^, ^36^ FGFR3 mutations are common in nonmuscular invasive BC and associated with favorable BC prognosis.^37^ P53, as an important tumor suppressor gene, is mutated in more than 50% of human malignant tumors, thus promoting the occurrence and development of tumors.^38^ TTN is a gene encoding sarcomere, and there has been no report on BC. The mutation of these genes not only plays an important role in the carcinogenesis of BC, but also participates in the transformation of different subtypes and immune escape.

Cancer cells and immune system form a complex immune network between their struggle, and which is also the premise and guarantee of tumor immune escape. The maladjustment of immune checkpoint PD‐1 is one of the main mechanisms for tumor cells to achieve immune escape.^39^ However, the specific mechanisms in different tumors need further study. In this study, the 552 subtype‐related immune genes were divided into 6 modules by WGCNA analysis, among which the brown module was the most correlated with the subtype of BC. The KEGG pathways enrichment analysis shows that pathways associated with the immune subtypes are as follows, neuroactive ligand‐receptor interaction, cytokine‐cytokine receptor interaction, primary immunodeficiency, viral protein interaction with cytokine and cytokine receptor, natural killer cell‐mediated cytotoxicity, graft‐versus‐host disease, regulation of actin cytoskeleton, and MAPK, Rap1, Ras, IL‐17, ErbB, B‐cell receptor, and other signaling pathways. Of the enrichment results, Natural killer cell (NK)‐mediated cytotoxicity can regulate NK cells in tumor microenvironment and plays an important role in monitoring and controlling tumor.^40^, ^41^ The main activated receptor of NK killing early tumor cells is natural cytotoxicity receptors and NKG2D.^40^ B‐cell receptor plays a major role in maintaining immunotherapy and self‐tolerance. The existence of B cells and tertiary lymphoid structures in tumor tissues can enhance the effect of immunotherapy.^42^, ^43^ IL‐17‐ induced a variety of complicated factors and chemokines can promote the recruitment of a variety of immune cells, to play a role in immune promotion.^44^ MAPK pathway is one of the main downstream pathways associated with IL‐17.^45^ These are the main pathways directly related to immunity, and other pathways also play important roles in tumor immune escape and deserve further study. In particular, regulation of actin cytoskeleton is one of the most interesting pathways associated with immune escape in BC. A study shows that ATF3 could inhibit the metastasis of BC cells by upregulation GSN‐mediated actin remodeling,^46^ but the relationship between bladder muscle and immune is not clear. In this study, TTN, as a gene encoding sarcomere, was the most common mutant gene in Cluster 2, which also indicates the importance of muscle tissue in BC.

This study has some limitations. First of all, the samples of each subtype are relatively small in the training and validation sets, and it was easy to miss some relatively rare immune subtypes of BC. Second, in order to explore the immune subtypes comprehensively, more clinical and demographic characteristics of BC patients should be included. In future studies on BC immune subtypes, a larger sample analysis should be carried out to find some rare clinical immune subtypes. Moreover, research on the immune escape‐driven mechanism and the transformation of immune subtypes during the carcinogenesis of BC may have more important findings.

In conclusion, the current study suggests that the immune phenotypes of BC could be classified into 4 molecular subtypes with potential immune escape mechanisms in BC. Patients of different subtypes have significant differences in the immune checkpoint molecules, HLA genes, gene mutations, immune cells, and prognostic, etc. Specific functional pathways or gene mutations may drive the formation of microenvironment immune subtypes. In addition, through the construction of decision tree model, the subtype of BC patients with high‐throughput sequencing could be accurately predicted. These results may provide guidance for developing novel strategies of immunotherapy in BC, and to a certain extent, prove the feasibility of predicting immune response via immune molecular subtypes.

## CONFLICT OF INTEREST

The authors declare no financial conflict of interest.

## AUTHOR CONTRIBUTIONS

The study conception and design were performed by J.C. J.L. and JT. Material preparation, data collection, and analysis were performed by J.C., J.L., X.Y., P.L., and Z.Y. The first draft of the manuscript was written by J.C., D.H., and L.W. All authors commented on previous versions of the manuscript. All authors read and approved the final manuscript.

## ETHICS STATEMENT

Bioinformatics analysis and publicly available datasets were used in this study, and ethical approval is not required.

## Supporting information

Table S1

Table S2

Table S3

Table S4

Table S5

## Data Availability

Publicly available datasets were analyzed in this study. The results published here are mainly based upon data generated by TCGA (https://www.cancer.gov/tcga) and GEO (www.ncbi.nlm.nih.gov/gds/) database.
